# Prognostic Values of G-Protein Mutations in Metastatic Uveal Melanoma

**DOI:** 10.3390/cancers13225749

**Published:** 2021-11-17

**Authors:** Mizue Terai, Ayako Shimada, Inna Chervoneva, Liam Hulse, Meggie Danielson, Jeff Swensen, Marlana Orloff, Philip B. Wedegaertner, Jeffrey L. Benovic, Andrew E. Aplin, Takami Sato

**Affiliations:** 1 Department of Medical Oncology, Sidney Kimmel Cancer Center, Thomas Jefferson University, Philadelphia, PA 19107, USA; liam.hulse@jefferson.edu (L.H.); meggie.danielson@jefferson.edu (M.D.); marlana.orloff@jefferson.edu (M.O.); takami.sato@jefferson.edu (T.S.); 2 Department of Pharmacology and Experimental Therapeutics, Sidney Kimmel Cancer Center, Thomas Jefferson University, Philadelphia, PA 19107, USA; ayako.shimada@jefferson.edu (A.S.); inna.chervoneva@jefferson.edu (I.C.); 3 Molecular Genetics, Caris Life Sciences, Phoenix, AZ 85040, USA; jswensen@carisls.com; 4 Department of Biochemistry and Molecular Biology, Sidney Kimmel Cancer Center, Thomas Jefferson University, Philadelphia, PA 19107, USA; philip.wedegaertner@jefferson.edu (P.B.W.); jeffrey.benovic@jefferson.edu (J.L.B.); 5 Department of Cancer Biology, Sidney Kimmel Cancer Center, Thomas Jefferson University, Philadelphia, PA 19107, USA; andrew.aplin@jefferson.edu

**Keywords:** uveal melanoma, metastasis, metastatic uveal melanoma, survival, *GNA11*, *GNAQ*, Q209P, Q209L, *BAP1*, *SF3B1*

## Abstract

**Simple Summary:**

Uveal melanoma (UM) is the most common primary intraocular malignancy in adults. More than 90% of UMs harbor mutually exclusive activating mutations in G-proteins. The mutations are early events in UM development and considered to be driver mutations in carcinogenesis. Even after treatment of primary uveal melanoma, up to 50% of patients subsequently develop recurrence, predominantly in the liver. *GNAQ* mutations are not reported to be correlated to survival, while the mutations in *GNA11* are reported more frequently in metastatic UM. We investigated the correlation of survival after development of metastasis (Met-to-Death) of metastatic uveal melanoma (MUM) patients with *GNA11* and *GNAQ* mutations. We identified that MUM with mutation patterns of Q209P vs. Q209L in *GNA11* and *GNAQ* might predict survival of MUM patients.

**Abstract:**

Uveal melanoma is the most common primary ocular malignancy in adults, characterized by gene mutations in G protein subunit alpha q (*GNAQ*) and G protein subunit alpha 11 (*GNA11*). Although they are considered to be driver mutations, their role in MUM remains elusive. We investigated key somatic mutations of MUM and their impact on patients’ survival after development of systemic metastasis (Met-to-Death). Metastatic lesions from 87 MUM patients were analyzed by next generation sequencing (NGS). *GNA11* (41/87) and *GNAQ* (39/87) mutations were most predominantly seen in MUM. Most *GNA11* mutations were Q209L (36/41), whereas *GNAQ* mutations comprised Q209L (14/39) and Q209P (21/39). Epigenetic pathway mutations *BAP1* (42/66), *SF3B1* (11/66), *FBXW7* (2/87), *PBRM1* (1/66), and *SETD2* (1/66) were found. No specimen had the EIF1AX mutation. Interestingly, Met-to-Death was longer in patients with *GNAQ* Q209P compared to *GNAQ/GNA11* Q209L mutations, suggesting the difference in mutation type in *GNAQ/GNA11* might determine the prognosis of MUM. Structural alterations of the GNAQ/GNA11 protein and their impact on survival of MUM patients should be further investigated.

## 1. Introduction

Uveal melanoma (UM) is the most common primary intraocular malignancy in adults with incidence rate of 5.2 cases per million per year in the United States [1]. Among all melanomas involving the eye, 83% arise from uvea (UM), 5% from conjunctiva, and 10% from other sites [2]. Despite the common embryologic origin of cutaneous and uveal melanocytes, UM has many differing epidemiologic, prognostic, biological features and molecular mechanisms which differ from cutaneous melanoma [3]. For instance, the majority of cutaneous melanoma harbor mutations of B-Raf proto-oncogene, serine/threonine kinase (*BRAF*), NRAS proto-oncogene, GTPase (*NRAS*) and neurofibromin 1 (*NF1*) as well as loss of cyclin dependent kinase inhibitor 2A (*CDKN2A*) encoding P16NK4a. On the other hand, uveal melanoma rarely harbors these abnormalities. [4,5,6].

Regardless of successful therapy of primary UM, up to 50% of patients subsequently develop systemic recurrence, especially in the liver via the hematogenous route. A bimodal pattern of the mortality displays the first peaks at 2–3 years and the second surge at 8–9 years after enucleation [7]. After development of hepatic metastasis, the median survival of patients is reported to be 12 to 17 months [8].

G protein-coupled receptor (GPCR) pathway mutations in UM has been well documented. Mutations in G protein subunit alpha q (*GNAQ*) and G protein subunit alpha 11 (*GNA11*) are the most common in UM and considered to be driver mutations in carcinogenesis [9,10]. Also reported in UM are mutations in cysteinly leukotriene receptor 2 (*CYSLTR2*) or phospholipase C beta 4 (*PLCB4*) which are located directly upstream or downstream of the G protein [11,12]. In general, these mutations are mutually exclusive and seen as somatic mutations. Approximately 90% of UM reportedly possesses mutually exclusive *GNA11* and *GNAQ* mutations [9,13]. Mutations are frequently seen at the conserved catalytic glutamine (Q209 in Gα_q_, exon 5) replaced by either Proline, P, or Leucine, L, which leads to GTPase function deficiency and constitutive activation. Less frequently, mutations were found at position 183 (exon 4) replacing Arginine, R. *GNA11* and *GNAQ* Q209 mutations are considered to send stronger signals to downstream pathways compared to R183 mutations [9,14]. These oncogenic mutations trigger a wide range of cell signaling cascades including the mitogen-activating protein kinases (MAPK), phosphoinositide 3-kinase/serine/threonine protein kinase (PI3K/AKT), and yes-associated protein/transcriptional co-activator with PDZ-binding motif (YAP/TAZ) pathways [15,16]. These mutations arise early in tumor evolution and may promote tumor progression [17]. Although these mutations are the predominant pathways for the development of UM, they are reportedly not associated with overall survival of UM patients [18]. In particular, *GNAQ* mutations were not correlated with disease-free survival, while the mutations in *GNA11* have been reported more frequently in metastatic UM (MUM) [19,20]. It is speculated that mutations in UM with *GNA11* mutations may be more aggressive than those with *GNAQ* mutations.

UM may have secondary somatic mutations affecting BRCA1 associated protein 1 (*BAP1*), splicing factor 3b subunit 1 (*SF3B1*), serine and arginine rich splicing factor 2 (*SRSF2*), or eukaryotic translation initiation factor 1A X-linked (*EIF1AX*), which tend to occur exclusively from each other [13,21]. These secondary mutations determine the metastatic potential of UM cells. For example, UM with *BAP1* alterations tends to develop systemic recurrence earlier compared to those with *SF3B1* mutations [22,23]. Tumor cells which harbor *EIF1AX* mutation tend not to metastasize [23].

These previous studies focused on time from initial diagnosis and treatment of primary UM to development of systemic recurrence or death. These studies did not address prognostic factors to determine time from diagnosis of metastasis to death (Met-to-Death). Here in this study, we aim to identify the correlation of Met-to-Death in MUM patients with commonly seen mutations in MUM. We identified that differences in mutation patterns (Q209P vs. Q209L) in *GNAQ* and *GNA11*, rather than *GNAQ* and *GNA11* themselves, might predict the survival of MUM patients.

## 2. Subject and Methods

### 2.1. Patients and Clinical Data

Tumor samples were collected and profiled from patients with MUM between the year 2013 and 2018 in this retrospective analysis. This study was approved by the Institutional Review Board (IRB) at Thomas Jefferson University [IRB#18D-183]. Clinical data were obtained from their medical records.

### 2.2. Tumor Tissue Samples

Tissue specimens from metastatic uveal melanoma patients were retrieved in paraffin-embedded archival core biopsy or surgically removed specimens for mutation analysis. Molecular profiling studies were done at Caris Life Sciences (Headquarters, Irving, TX, USA), except one specimen which was done at Foundation Medicine (Headquarters, Cambridge, MA, USA). In terms of molecular analysis done by Caris Life Sciences, either formalin-fixed paraffin-embedded tissue block or formalin-fixed paraffin-embedded 10 unstained tissue slides that could be enriched to a minimum of 20% tumor by microdissection were sent to Caris Life Sciences. Detailed information on molecular profiling technology is available from the following website (https://www.carislifesciences.com/molecular-profiling-technology (accessed on 28 September 2021)). In brief, tumor DNA was extracted from the tumor specimens obtained by microdissection of the tumor area under the supervision of board-certified pathologists. Tumor samples were analyzed by next-generation sequencing (NGS) of exons using either TruSeq Amplicon 48 Gene Cancer Panel with MiSeq system (Illumina, San Diego, CA, USA) or 592 cancer-relevant genes panel (SureSelect XT, Agilent, Santa Clara, CA, USA) with the NextSeq instrument (Illumina). The overall average depth of coverage was typically >1000×. Variants were called based on a combination of coverage and depth using a sliding scale. The minimum possible variant frequency was approximately 5%. The minimum depth for a called variant was 100×. If coverage fell below 100× in any region of a gene, the entire gene was called “Indeterminate.” Amplification analysis was performed in samples tested by the 592 gene panel. Genes with at least six copies were called amplified. The data for the analysis in this study were obtained from a clinical laboratory system of Thomas Jefferson University that stores all laboratory results ordered by physicians.

### 2.3. Statistical Analysis

We investigated the correlation between somatic mutations in MUM specimens and survival of patients after development of systemic metastasis. The endpoint of survival analysis was from metastasis to death (Met-to-Death).

Patient characteristics were summarized with frequency counts and percentages for categorical variables and median and interquartile range (IQR) for continuous variables.

For unadjusted comparison of patient characteristics by gene mutation groups, Fisher’s exact test was used for categorical variables, and the Kruskal-Wallis test was used for continuous variables.

The Kaplan-Meier (K-M) survival curves were used to estimate the overall survival (OS) from metastasis and the corresponding median survival time by patient characteristics. Log-rank tests were used to compare the K-M curves.

Univariable Cox proportional hazards models was used to assess the association between survival from metastasis and continuous clinical-pathological factors: age, and time from the primary treatment to the metastases. Multivariable Cox proportional hazards model was used to evaluate the effect of gene mutation type on OS while controlling for other significant predictors of OS. Death due to metastasis was considered an event. All the analyses were performed with SAS 9.4 (SAS Institute Inc., Cary, NC, USA).

## 3. Results

### 3.1. Tumor and Patient Characteristics

We submitted 102 samples to commercial companies for analysis; 15 samples were indeterminated for mutation analysis. A total of 87 patients were analyzed for somatic mutations in their metastatic specimens. Of 87 patients, 21 patients were analyzed by NGS with 48 selected cancer genes including *GNA11*, *GNAQ* and *FBXW7*, and 66 patients were analyzed for mutations by NGS with 592 cancer genes including *BAP1* and *SF3B1* mutations as well as *MYC* amplification (Supplement Table S1). Median survival time after metastasis was 25 months with a range of 1–137 months. Metastatic specimens were collected within 1 year from diagnosis of metastasis in 72 patients, while 9 specimens were obtained 1–2 years after initial diagnosis of metastasis and 6 specimens were obtained more than 2 years after initial diagnosis of metastasis. No treatment targeting *GNAQ/GNA11* mutations was given to any of these patients.

At the end of follow-up, 72 patients died including one unexpected non-cancer event; 2 patients were lost to follow-up; and 13 patients were alive (total 87 patients). There were 46 males and 41 females (Table 1). Median ages at diagnosis of primary and metastatic UM were 58 and 60 years of age, respectively. Most patients were treated with radioactive plaque for their primary UM (74.7%) and 90.8% of patients received a liver-directed therapy in the first and/or second treatment option of metastatic UM [24,25,26,27]. Those diagnosed with the first metastatic lesion in this patient cohort were mostly of the liver, lung and omentum, 84/87 (96.5%), 2/87, (2.3%), 1/87, (1.2%), respectively. Those patients who were diagnosed with only extrahepatic metastases initially had subsequently developed liver metastases (3/3, 100%).

### 3.2. Frequency of GNA11 and GNAQ Mutations in Metastatic Uveal Melanoma

Mutations in *GNA11* and *GNAQ* are considered to be driver mutations that lead to constitutive activation of GPCR signaling. We first assessed these known UM driver mutations. The frequent alteration of *GNAQ* and *GNA11* were identified as nearly mutually exclusive in 81 of 87 patients (92.0%) (Table 2). Mutually exclusive *GNA11* mutations were found in 41/87 (47.1%) tumors, while *GNAQ* mutations were found in 39/87 tumors (44.8%). One case (Case 23) harbored two simultaneous mutations of *GNA11* Q209L in exon 5 and *GNAQ* T96S in exon 2, which might indicate the tumor specimen contained a heterozygous population of tumor cells (Supplement Table S2). Six out of 87 tumors were not found to have either *GNA11* or *GNAQ* mutation. As previously reported exon 5 of *GNA11* and *GNAQ* genes, which contains the hotspot mutation leading to the most frequent alteration of the Q209 amino acid, was identified in 74 of 87 patients (85.1%) (Table 2). Frequently, mutations were seen at the position 209 glutamine (Q) to either proline (P) or leucine (L). Of those with *GNA11* mutations, the Q209L mutation was found in 37 of these 42 cases (88.1%) while Q209P mutation was found in one case (Case 52) (2.4%). One specimen showed the Q209M mutation (Case 75), one case (Case 74) harbored both the R183C and V344M mutations in *GNA11*, and two cases had a single mutation at R183C. One specimen had *GNA11* Q209L mutation as well as *GNAQ* T96S mutation (Case 23) (Supplement Table S2).

Of those with *GNAQ* mutation, the Q209L mutation was found in 14 of these 40 cases (35.0%) while Q209P mutation was found in 21 of 40 cases (52.5%). Two MUM specimens harbored a single mutation at R183Q in exon 4 (2/39, 5.1%). One showed a single mutation at G48L in exon 2 (Case 79). One specimen showed simultaneous mutations of R183Q in exon 4 and R338H in exon 7 (Case 80). One specimen (Case 23) had *GNAQ* T96S mutation as well as *GNA11* Q209L mutation. (Table 2, Supplement Table S2). As expected, the mutations in *GNA11* and *GNAQ* were almost mutually exclusive except for one specimen. These results show that double mutations seem to be rare events in MUM as previously reported in primary UM [9,13].

### 3.3. Other Somatic Mutations

Among 87 MUM specimens, 66 specimens were analyzed for target-captured deep sequencing of 592 cancer genes. Other mutations found in MUM specimens were mostly related to epigenetic pathways including *BAP1* (42/66; 63.6%), *SF3B1* (11/66; 16.6%), F-box and WD repeat domain containing 7 (*FBXW7*) (2/87; 2.3%), protein polybromo-1 (*PBRM1*) (1/66; 1.5%), and SET domain containing 2, histone lysine methyltransferase (*SETD2*) (1/66; 1.5%) (Supplement Tables S1 and S2). One case (Case 80) harbored simultaneous alterations of *BAP1* and the mutation of *SF3B1*. In addition, *MYC* amplifications were assessed and 13 out of 66 specimens (19.7%) were positive for *MYC* amplifications (Supplement Table S1). No specimen had *EIF1AX* mutation. *NRAS* and *BRAF* mutations, which are commonly seen as driver genes in cutaneous melanoma, were not found either.

Location of *BAP1* alterations were variable. *SF3B1* mutations including R625C, R625H, R625G, R625L, N626Y, and G742D were detected. *SF3B1* mutations were almost always mutually exclusive of the presence of *BAP1* alteration in tumor specimens except one specimen.

Two tumors (Cases 34 and 70) had *FBXW7* mutations, which has been reported as a tumor suppressor gene [28,29]; one sample carried the mutation in exon 12, c.1856-2A > G; and another sample carried exon 4, c.585-1G > T. One tumor (Case 34) had a mutation (exon 11, c.996-1G > T) of *PBRM1*, which is the second most common tumor suppressor gene in kidney cancer. The other tumor (Case 73) had a mutation (exon 1, c.68_71 + 1delCTGAG) of *SETD2*, a histone methyltransferase that mediates trimethylation of lysine 36 on Histone 3 (Supplement Table S2).

### 3.4. Survival Analysis of Met-to-Death

To examine the possibility for showing a difference in clinical characteristics of tumors with different mutations, we analyzed the Met-to-Death between the mutation of proline (P) and leucine (L) at the position of 209 in *GNA11* or *GNAQ*.

Among 74 patients whose tumors harbored Q209 mutations, 69 patients were analyzed for their survival data. One specimen with mutations in both *GNAQ* and *GNA11* and specimens with *GNA11* Q209P (*n* = 1) and Q209M (*n* = 1) were excluded from this statistical analysis due to small sample size for statistical justification. Two patients with *GNAQ* Q209P were also removed from survival analysis due to patients lost in follow-up. Table 3 summarizes the patient characteristics of each group. For unadjusted comparison, there were no statistically significant associations between gene mutation types and patient characteristics (Table 3). Similar proportions of patients with and without BAP1 alterations and *SF3B1* mutations were observed across different *GNA11*/*GNAQ* mutation types. Other potential confounding factors such as M stage, treatment after metastasis, and time from primary eye treatment to development of metastasis were comparable.

**Figure 1:**
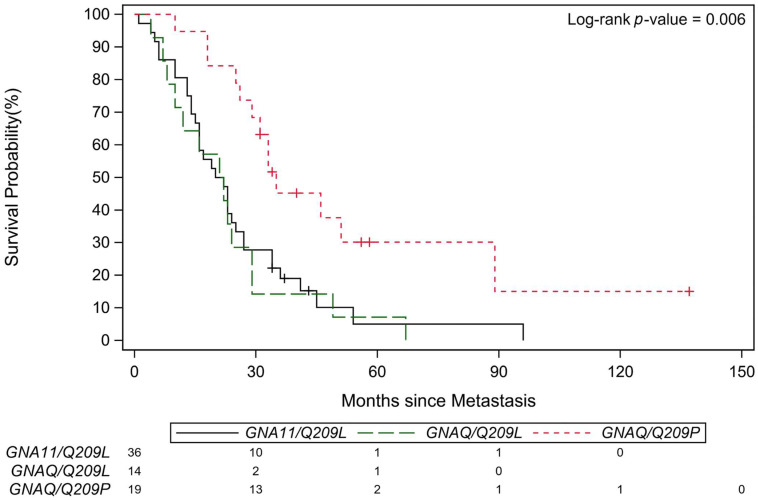
The Kaplan-Meier Curves for Met-to-Death OS. Kaplan-Meier Curves showing patients carrying tumor mutations with Q209L of *GNA11*, Q209L of *GNAQ* and Q209P of *GNAQ*. The red line represents Q209P of *GNAQ* (*n* = 19), the green line represents Q209L of *GNAQ* (*n* = 14) and the black line represents Q209L mutation of *GNA11* (*n* = 36).

We then analyzed the association of the patient characteristics and the time from Met-to-Death. Kaplan Meier survival (K-M) curve showed patients with *GNAQ* Q209P mutant tumors had a more favorable outcome than patients with *GNA11* Q209L and *GNAQ* Q 209L mutant tumors after development of metastasis (*n* = 69, Log-rank test, *p* = 0.006, Table 4 and Figure 1). The univariable Cox models revealed patients with *GNA11* Q209L mutant tumors or *GNAQ* Q209L mutant tumors showed shorter median survival (Met-to-Death) than patients with *GNAQ* Q209P mutant tumors. The median survival (Met-to-Death) was 21 months (95% CI: 15–25) for *GNA11* Q209L, 21.5 months (95% CI: 8–29) for *GNAQ* Q209L, and 35 months (95% CI: 26–89) for *GNAQ* Q209P, respectively (*p* = 0.006) (Table 4).

There was a difference in Met-to-Death OS by *BAP1* status. Patients with *BAP1* alterations showed shorter median survival than those without *BAP1* alterations (Table 4). Although the sample size of patients with known *SF3B1* status was small (*n* = 50), MUM patients whose tumors had *SF3B1* mutations (*n* = 7) had the tendency to live longer [median survival of 89 months (95% CI: 13–96, *n* = 7)] than those who did not have this mutation [median survival of 23 months (95% CI: 19–29), *n* = 43)] (*p* = 0.011) (Table 4). Based on this observation, we reanalyzed the survival between *BAP1* alterations and non *BAP1* alterations. All patients with *SF3B1* mutations were excluded from the non *BAP1* alteration group for this analysis. After removing patients with *SF3B1* mutations, Met-to-Death OS analysis showed no statistical difference between patients with *BAP1* alteration (*n* = 31) and without *BAP1* alteration (*n* = 12) (*p* = 0.845) (Supplement Table S3). Additionally, we observed about 4% increase in hazard of death per each additional year of age at eye diagnosis (Hazard Ratio (HR) = 1.04, 95% CI: 1.02–1.07; *p* < 0.001) and age at metastasis (HR = 1.04, 95% CI: 1.01–1.06; *p* = 0.004) (Table 4). These were consistent findings as reported by Seeder, et al. [8].

Multivariable Cox models were also performed to evaluate the effect of gene mutation types on Met-to-Death OS with adjusting age at metastasis and log-transformed time from primary treatment to metastasis (Table 5). The analysis revealed that patients with *GNA11* Q209L mutant tumors or *GNAQ* Q209L mutant tumors had shorter Met-to-Death OS as compared to patients with *GNAQ* Q209P mutant tumors (Hazard Ratio (HR); 3.42, 95% CI: 1.68–6.96, *p* = 0.001 for *GNA11* Q209L vs. *GNAQ* Q209P, and HR = 3.08, 95% CI: 1.35–7.04, *p* = 0.008 for *GNAQ* Q209L vs. *GNAQ* Q209P). The comparison of Hazard between patients with *GNAQ* Q209L mutant tumors and patients with *GNA11* Q209L mutant tumors did not show a significant difference (HR = 1.11, 95% CI: 0.57–2.15; *p* = 0.759) (Table 5). Older age at metastasis diagnosis was independently associated with a higher hazard of death (HR = 1.04, 95% CI: 1.01–1.06; *p* = 0.005). Additionally, longer time from the initial eye treatment to metastasis was associated with lower hazard of death (HR = 0.79, 95% CI: 0.63–0.99; *p* = 0.041).

Tumors with *BAP1* alterations in UM is a consistent finding for a poor prognosis marker [30]. To confirm the result of the multivariable Cox model in Table 5, we also examined the patients with tumor-analyzing *BAP1* status (*n* = 50). We excluded 19 patients with *GNAQ* or *GNA11* at Q209 mutant tumors who had no data on *BAP1* status. Due to sample size limitation, *BAP1* wild type specimens with *SF3B1* mutations were not excluded from the *BAP1* alteration negative population in this analysis. Although patients with *BAP1* altered tumors tended to show poorer Met-to-Death survival than patients with *BAP1* wild type tumors, the result was not statistically significant (HR = 1.62, 95% CI: 0.75–3.60; *p* = 0.214) (Supplement Table S4). Meanwhile, controlling for *BAP1* status, patients with *GNA11* Q209L mutant tumors (*n* = 29) or *GNAQ* Q209L mutant tumors (*n* = 13) still had a poor Met-to-Death survival as compared to patients with *GNAQ* Q209P mutant tumors (*n* = 16) (HR = 4.07, 95% CI: 1.62–10.23; *p* = 0.003 for *GNA11* Q209L, and HR = 3.69, 95% CI: 1.31–10.36, *p* = 0.0013 for *GNAQ* Q209L) (Supplement Table S4).

## 4. Discussion

In this study, we investigated the frequency of mutations in MUM specimens and the role of commonly mutated *GNA11*/*GNAQ* genes in survival after development of systemic metastasis (Met-to-Death). In 87 MUM patients, we showed that *GNA11* and *GNAQ* mutations were found in 47.1% and 44.8% of patients, respectively. This result was consistent with the analysis performed by other researchers in primary uveal melanoma [9,31], whereas Griewank et al. report on MUM showed more *GNA11* mutations than *GNAQ* mutations in MUM patients [20]. There also has been inconclusive discussion whether prognosis of UM tumors with GNA11 is poorer than that of UM tumors with *GNAQ* mutations. It is of note that glutamine (Q) at position 209 in *GNA11* was commonly replaced with leucine (L) in 97.3% of samples, compared to replacement with proline (P) in 2.7% of these samples [9,20]; therefore, potentially poor prognosis of *GNA11* mutated UM tumors might be due to dominant 209L mutation in *GNA11*. We tested this hypothesis and investigated Met-to-Death survival in patients with MUM after stratifying mutations at position 209 between *GNA11* and *GNAQ*. Interestingly, patients with Q209P in *GNAQ* mutant tumors significantly correlated with favorable prognosis after development of metastasis. Traditionally, the prediction of prognosis in UM patients has been based on the survival from the date of treatment of primary UM to their death. It has been reported that loss of Chromosome 3 and Chromosome 8q gains in primary UM tumor specimens have been shown to predict survival of UM patients [13,32,33]. In addition, the expression gene profile of class 2 is used for the prediction of prognosis [34]. Currently, other biological prognostic markers are explored with the expression of PRAME or autophagy related proteins in primary uveal melanoma specimens. The level of Beclin-1 expression on primary uveal melanoma correlated with a lower risk of metastasis and higher disease-free survival times [35]. On the other hand, the expression of PRAME identified increasing metastatic risk [36]. None of the above investigations was extended to the analysis on Met-to-Death and the factors to predict survival after development of metastasis remains to be investigated.

*GNA11* and *GNAQ* mutations were considered to occur early and represent initiating events in tumorigenesis [17]. It has been shown that the difference in *GNAQ* and *GNA11* mutations did not affect survival of UM patients after their treatment of primary UM. In this regard, it is rather interesting that our data showed substitutions Q209L vs. Q209P rather than G protein (GNAQ vs. GAN11) impacting the survival of UM patients with metastasis. The substitutions in Q209 might play a role in determining prognosis of MUM patients, especially Met-to-Death.

It remains to be investigated why UM patients with *GNAQ* Q209P mutant tumors showed favorable outcome after development of metastasis in our study. Since clinical teams does not make any treatment decision based on *GNAQ* and *GNA11* mutation status, there was no difference in their clinical stages and treatment approaches to these patients, including M stage and choice of treatments. There was no difference in frequency of *BAP1* or *SF3B1* mutations and time from primary eye treatment to development of systemic recurrence among MUM patients with Q209L and Q209P mutations. One of the possibilities is that Q209P mutant tumor may have a signature of higher immunological characteristics than Q209L mutant tumor. Immunogenicity of Q209P mutation might be different from that of Q209L. In this regard, Weeghel et al. reported Q209P or Q209L mutation does not have significant impact on the immunological characteristics of the tumors [37]. It is also possible that the main difference between Q209P mutant and Q209L mutant is that one may have unique structural properties that may impact its ability to bind different interacting partners such as G protein βγ subunits, and Q209P may have a distinct functional feature not shared by Q209L [38]. Different degrees and pathways of downstream signal transduction might result in resistance to treatments and contribute to difference in survival of MUM patients. Investigation on this possibility is underway in our group.

A more widely accepted concept is that UM patients with *BAP1* alterations have poor prognosis. Based on our data supported by our basic research experiments using *BAP1* altered cell lines, this might not be due to rapid growth of *BAP1* altered cells in metastasis. In point of fact, it is reported that BAP1-deficient UM cell lines generally are associated with extremely slow growth characteristics [39]. The distinct slow doubling time that has been seen in established cell lines *in vitro* has not represented patient outcome. In fact, impact of *BAP1* alteration on Met-to-Death is not clearly shown in our survival analysis excluding patients with *SF3B1* mutations. It is of note that the detection rate of *BAP1* alteration in our subjects is relatively lower than previously published data [5,20]. This is most likely due to differences in detection assay since inactivating *BAP1* alterations can be present anywhere along the gene body. The panel sequencings used for our analysis were *BAP1* exons assay which did not include intron sequences for *BAP1* gene. Furthermore, our NGS assay did not detect large deletions or duplications over ~100 bp. Further studies with more detailed *BAP1* gene analyses are needed to investigate the association of *BAP1* alteration with Met-to-Death. *BAP1* alterations might be an important factor for development of systemic recurrence; however, the role of *BAP1* alteration for rapid growth of metastasis or resistant mechanisms for treatments remain to be proven. In this regard, Szalai et al., reported the metastatic pattern might be different between patients with *BAP1* altered tumor and *SF3B1* mutant tumor. Time to clinically detectable metastases has different peaks among these two mutations. The earlier peaks appear to be associated with *BAP1* altered tumors and the later peak is associated with *SF3B1* mutated tumors [23]. Although the sample size is small and further investigation with a larger cohort is required, our data also indicate that the presence of *SF3B1* mutations might affect the survival of MUM patients (Met-to-Death). These data indicate that *BAP1* alterations may be a predictive factor for early systemic recurrence; however, it might not be a prognostic factor for MUM patients who already developed metastasis. Furthermore, future clinical trials might require the stratification of MUM patients based on the status of *SF3B1* mutations in addition to type of *GNAQ*/*GNA11* Q209 mutations (P vs. L).

Lastly, our data showed the amplification of *MYC* was determined in 19.7% of metastatic specimens (13/66). The progression of uveal melanoma has often seen with the amplification of chromosome 8q which is the common region of amplification found to range from 8q24.1 to 8q24.3 [40]. The proto-oncogene *MYC* is located at 8q24.12-q24.13 and is one candidate for the amplification at this site. Parrella et al., reported 70% of uveal melanoma detected extra copies of the region around the *MYC* locus by fluorescent in situ hybridization [41]. Later, Ehlers et al. analyzed the region of chromosome 8q with gene expression microarray analysis using Affymetrix Hu133A and B GeneChips. They reported that *DDEF1* gene, located at chromosome 8q24, was increased in 8q amplified tumor whereas MYC expression remained unchanged [42]. This indicates that *MYC* is not always amplified in 8q24 chromosome region in 8q-amplified but other oncogenes residing in chromosome 8q24 might play a role in tumor progression. Since numbers of specimens with *MYC* amplification were limited, we could not conduct detailed statistical analysis on the role of MYC on Met-to-Death. More studies at 8q24 loci are needed to explore the association of *MYC* amplification for tumor progression in uveal melanoma.

There are several limitations of this study. First of all, metastatic specimens were obtained more than 2 years from the diagnosis of metastasis in 6 of 87 patients. It is possible that mutational patterns of metastatic tumors had changed with previous treatments. Since there is no treatment given directly targeting *GNAQ*/*GNA11* mutations, we believe it is less likely that the frequency and pattern of *GNAQ*/*GNA11* mutations changed from the diagnosis of metastasis to the time of tumor specimen procurement in this study. Obviously, we do not exclude the possibility of differences in tumor characteristics between primary uveal melanomas and their metastasis.

Another potential confounding factor is the change in analysis methods during the study period. Of 87 patients, 21 patients were analyzed using TruSeq Amplicom 48 Gene Cancer Panel with the MiSeq system for *GNAQ* and *GNA11* mutations, while the rest were analyzed using 592 genes panel with the NexrSeq instrument for NGS assay. Since individual assays were validated for commercial use, we believe the results of *GNAQ*/*GNA11* mutation analysis in individual assays are reliable and consistent. We did not include *PLCB4* and *CYSLTR2* mutational status for our analysis since those data were not available for analysis in our dataset. Six specimens that had no *GNAQ* and *GNA11* mutations might be identified to have either *PLCB4* or *CYSLTR2*.

In terms of *BAP1* alteration analysis, it is possible that our assays underestimate the frequency of dysfunctional BAP1 in tumors since we did not check the intron sequences for *BAP1* gene. Since most tissue specimens were exhausted for NGS assay, we were not able to check the expression of BAP1 protein in tissue specimens. The sample size of this study is too small to reliably investigate the role of *BAP1* alternation and *SF3B1* mutations in survival of metastatic uveal melanoma patients and this remains to be investigated in future studies.

## 5. Conclusions

This clinical study indicates that MUM tumors with different mutations of Q209 in *GNAQ* and *GNA11* might have different characteristics in terms of survival and response to treatments after development of systemic metastasis. Among patients with MUM, Q209P mutation in their tumor specimens would have a more favorable prognosis than those with Q209L mutation after development of metastases. This might indicate a different signal transduction pattern between these two mutations and well-designed molecular studies should be considered to identify the difference between Q209P compared to *GNAQ*/*GNA11* Q209L mutant UM cells.

## Figures and Tables

**Table 1:** Demographic features of UM patients with metastasis.

Characteristic	Number
Number of Patients	87
Gender	
Male	46
Female	41
Age at primary eye diagnosis (years)	
Median (Range)	58 (21–88)
Male	59 (28–88)
Female	55 (21–77)
Location of primary uveal melanoma (%)	
Choroidal	67 (77.0)
Ciliary	20 (23.0)
Other	0
Treatment for primary uveal melanoma (%)	
Radiopaque	65 (74.7)
Enucleation	13 (14.9)
Others	7 (8.0)
Unknown	2 (2.2)
Age at metastasis diagnosis (years)	
Median (Range)	60 (24–88)
Male	60 (28–88)
Female	60 (24–78)
Metastatic site (at the diagnosis) (%)	
Liver	84 (96.5)
Lung	2 (2.3)
Omentum	1 (1.2)
M stage (%)	
M1a ≤ 3 cm	69 (79.3)
M1b 3.1–8.0 cm	14 (16.1)
M1c ≥ 8.1 cm	4 (4.6)
First and second treatments option at metastatic site (%)	
Liver-directed alone	41 (47.1)
Liver-directed + Systemic	38 (43.7)
Systemic alone	4 (4.6)
Recession	1 (1.1)
Unknown	3 (3.5)

**Table 2:** Frequency of mutations in the two major genes in metastatic uveal melanoma.

Mutation Status	N
*GNA11*	41 (47%)
Q209L	36
Q209P	1
Q209M	1
R183C	2
R183C, V344M	1
*GNAQ*	39 (45%)
Q209L	14
Q209P	21
R183Q	2
G48L	1
R183Q, R338H	1
Both *GNA11* and *GNAQ*	1 (1%)
*GNA11* Q209L, *GNAQ* T96S	1
No *GNA11* or *GNAQ*	6 (7%)

**Table 3:** Association between Patients Characteristics and the Type of Gene Mutation.

Characteristic		ALL (*n* = 69)	*GNA11*/Q209L (*n* = 36)	*GNAQ*/Q209L (*n* = 14)	*GNAQ*/Q209P (*n* = 19)	*p*-Value
Gender, *n* (%)						
	Female	31 (44.9)	19 (52.8)	5 (35.7)	7 (36.8)	0.391
	Male	38 (55.1)	17 (47.2)	9 (64.3)	12 (63.2)	
Primary Site, *n* (%)						
	Choroidal	54 (78.3)	26 (72.2)	11 (78.6)	17 (89.5)	0.373
	Ciliary	15 (21.7)	10 (27.8)	3 (21.4)	2 (10.5)	
Age at Primary Dx, median (IQR)						
		58.0 (50.0, 66.0)	59.0 (48.5, 66.5)	61.5 (57.0, 77.0)	54.0 (49.0, 61.0)	0.133
Age at Metastasis, median (IQR)						
		60.0 (54.0, 69.0)	60.0 (51.0, 69.0)	65.0 (60.0, 78.0)	59.0 (52.0, 63.0)	0.074
Metastasis site, *n* (%)						
	Liver	68 (98.6)	36 (100.0)	13 (92.9)	19 (100.0)	0.203
	Omentum	1 (1.4)	0 (0.0)	1 (7.1)	0 (0.0)	
Months from Primary Tx to Met, median (IQR)						
		25.0 (11.0, 44.0)	26.0 (11.0, 37.0)	27.0 (15.0, 57.0)	25.0 (14.0, 55.0)	0.809
*BAP1*—Mutation, *n* (%)						
	Yes	31 (44.9)	15 (41.7)	7 (50.0)	9 (47.4)	1.000
	No	19 (27.5)	10 (27.8)	4 (28,6)	5 (26.3)	
	N/A	19 (27.5)	11 (30.6)	3 (21.4)	5 (26.3)	
*SF3B1*—Mutation, *n* (%)	Yes	7 (10.1)	3 (8.3)	1 (7.1)	3 (15.8)	0.661
	No	43 (62.3)	22 (61.1)	10 (71.4)	11 (57.9)	
	N/A	19 (27.5)	11 (30.6)	3 (21.4)	5 (26.3)	
M stage (tumor size in Liver), *n* (%)						
	≤3 cm	53 (76.8)	28 (77.8)	8 (57.1)	17 (89.5)	0.209
	3.1–8.0 cm	12 (17.4)	5 (13.9)	5 (35.7)	2 (10.5)	
	≥8.1 cm	4 (5.8)	3 (8.3)	1 (7.1)	0 (0.0)	
Tx after metastasis (Tx1 + 2), *n* (%)						
	Liver direct Tx + Systemic Tx	31 (45.6)	17 (47.2)	5 (38.5)	9 (47.4)	0.730
	Liver direct therapy	33 (48.5)	17 (47.2)	7 (53.8)	9 (47.4)	
	Recession	1 (1.5)	0 (0.0)	1 (7.7)	0 (0.0)	
	Systemic Tx	3 (4.4)	2 (5.6)	0 (0.0)	1 (5.3)	

**Table 4:** Association between Patients Characteristics and Survival from Metastasis.

Characteristic		*N* (%)	Median OS (95% CI)	*p*-Value
Gender, *n* (%)				
	Female	31 (44.9)	31.0 (20.0, 41.0)	0.122
	Male	38 (55.1)	22.5 (15.0, 26.0)	
Primary Dx, *n* (%)				
	Choroidal	54 (78.3)	25.5 (20.0, 33.0)	0.331
	Ciliary	15 (21.7)	21.0 (13.0, 25.0)	
Met Dx site, *n* (%)				
	Liver	68 (98.6)	23.5 (19.0, 29.0)	0.387
	Omentum	1 (1.4)	67.0 *	
Gene Mutation, *n* (%)				
	*GNA11*/Q209L	36 (52.2)	21.0 (15.0, 25.0)	0.006
	*GNAQ*/Q209L	14 (20.3)	21.5 (8.0, 29.0)	
	*GNAQ*/Q209P	19 (27.5)	35.0 (26.0, 89.0)	
*BAP1*—Mutation, *n* (%)				
	Yes	31 (62.0)	25.0 (19.0, 31.0)	0.040
	No	19 (38.0)	36.0 (13.0, 89.0)	
		11		
*SF3B1*—Mutation, *n* (%)	Yes	7 (14.0)	89.0 (13.0, 96.0)	0.011
	No	43 (86.0)	23.0 (19.0, 29.0)	
M stage (tumor size in Liver), *n* (%)				
	≤3 cm	53 (76.8)	25.0 (22.0, 33.0)	0.102
	>3 cm	16 (23.2)	12.0 (7.0, 26.0)	
Tx after metastasis ^#^ (Tx1 + 2), *n* (%)				
	Liver direct Tx + Systemic Tx	31 (44.9)	33.0 (23.0, 45.0)	0.104
	Liver direct therapy	33 (47.8)	18.0 (14.0, 26.0)	
	Recession	1 (1.4)	23.0 *	
	Systemic Tx	3 (4.3)	13.0 *	
	Unknown	1		
			HR (95% CI)	
Age at Primary Dx				
			1.04 (1.02, 1.07)	<0.001
Age at Metastasis				
			1.04 (1.01, 1.06)	0.004
Months from Primary Tx to Met (log-transformed)				
			0.85 (0.69, 1.03)	0.101

* The 95% CI is not estimable due to the small number of observations. ^#^ Tx1 + 2 = First treatment + Second treatment.

**Table 5:** Results from the main multivariable Cox Model for OS from Met to Death.

Factor	HR	(95% CI)	*p*-Value
Gene Mutation			0.003
*GNA11*/Q209L vs. *GNAQ*/Q209P	3.42	(1.68, 6.96)	0.001
*GNAQ*/Q209L vs. *GNAQ*/Q209P	3.08	(1.35, 7.04)	0.008
*GNA11*/Q209L vs. *GNAQ*/Q209L	1.11	(0.57, 2.15)	0.759
Age at Metastasis (Continuous)	1.04	(1.01, 1.06)	0.005
Time from Primary Tx to Metastasis (log-transformed)	0.79	(0.63, 0.99)	0.041

## Data Availability

The data presented in this study are available in the supplementary material. The further data that support the findings of this study are available from the corresponding author upon reasonable request.

## Supplementary Materials

The following are available online at https://www.mdpi.com/article/10.3390/cancers13225749/s1, Table S1: Frequency of Mutations in Metastatic Uveal Melanoma Tissues, Table S2: Percentage of the Estimated Tumor DNA in Specimens and Frequency of Genetic Variants, Table S3: Association between BAP1 Mutation Status and Survival from Metastasis after Removing Patients with *SF3B1* Mutations (*n* = 43), Table S4: Results from the Multivariable Cox Model for OS from Met to Death in 50 Patients with Known *BAP1* Alteration status.

## References

[1] Aronow M.E., Topham A.K., Singh A.D. (2018). Uveal Melanoma: 5-Year Update on Incidence, Treatment, and Survival (SEER 1973-2013). Ocul. Oncol. Pathol..

[2] Kaliki S., Shields C.L. (2017). Uveal melanoma: Relatively rare but deadly cancer. Eye.

[3] van der Kooij M.K., Speetjens F.M., van der Burg S.H., Kapiteijn E. (2019). Uveal Versus Cutaneous Melanoma; Same Origin, Very Distinct Tumor Types. Cancers.

[4] Chacon M., Pfluger Y., Angel M., Waisberg F., Enrico D. (2020). Uncommon Subtypes of Malignant Melanomas: A Review Based on Clinical and Molecular Perspectives. Cancers.

[5] Karlsson J., Nilsson L.M., Mitra S., Alsen S., Shelke G.V., Sah V.R., Forsberg E.M.V., Stierner U., All-Eriksson C., Einarsdottir B. (2020). Molecular profiling of driver events in metastatic uveal melanoma. Nat. Commun..

[6] Russo D., Di Crescenzo R.M., Broggi G., Merolla F., Martino F., Varricchio S., Ilardi G., Borzillo A., Carandente R., Pignatiello S. (2020). Expression of P16INK4a in Uveal Melanoma: New Perspectives. Front. Oncol..

[7] Demicheli R., Fornili M., Biganzoli E. (2014). Bimodal mortality dynamics for uveal melanoma: A cue for metastasis development traits?. BMC Cancer.

[8] Seedor R.S., Eschelman D.J., Gonsalves C.F., Adamo R.D., Orloff M., Amjad A., Sharpe-Mills E., Chervoneva I., Shields C.L., Shields J.A. (2020). An Outcome Assessment of a Single Institution’s Longitudinal Experience with Uveal Melanoma Patients with Liver Metastasis. Cancers.

[9] Van Raamsdonk C.D., Griewank K.G., Crosby M.B., Garrido M.C., Vemula S., Wiesner T., Obenauf A.C., Wackernagel W., Green G., Bouvier N. (2010). Mutations in GNA11 in uveal melanoma. N. Engl. J. Med..

[10] Shoushtari A.N., Carvajal R.D. (2014). GNAQ and GNA11 mutations in uveal melanoma. Melanoma Res..

[11] Moore A.R., Ceraudo E., Sher J.J., Guan Y., Shoushtari A.N., Chang M.T., Zhang J.Q., Walczak E.G., Kazmi M.A., Taylor B.S. (2016). Recurrent activating mutations of G-protein-coupled receptor CYSLTR2 in uveal melanoma. Nat. Genet..

[12] Johansson P., Aoude L.G., Wadt K., Glasson W.J., Warrier S.K., Hewitt A.W., Kiilgaard J.F., Heegaard S., Isaacs T., Franchina M. (2016). Deep sequencing of uveal melanoma identifies a recurrent mutation in PLCB4. Oncotarget.

[13] Robertson A.G., Shih J., Yau C., Gibb E.A., Oba J., Mungall K.L., Hess J.M., Uzunangelov V., Walter V., Danilova L. (2017). Integrative Analysis Identifies Four Molecular and Clinical Subsets in Uveal Melanoma. Cancer Cell.

[14] Shirley M.D., Tang H., Gallione C.J., Baugher J.D., Frelin L.P., Cohen B., North P.E., Marchuk D.A., Comi A.M., Pevsner J. (2013). Sturge-Weber syndrome and port-wine stains caused by somatic mutation in GNAQ. N. Engl. J. Med..

[15] Park J.J., Diefenbach R.J., Joshua A.M., Kefford R.F., Carlino M.S., Rizos H. (2018). Oncogenic signaling in uveal melanoma. Pigment. Cell Melanoma Res..

[16] Chua V., Lapadula D., Randolph C., Benovic J.L., Wedegaertner P.B., Aplin A.E. (2017). Dysregulated GPCR Signaling and Therapeutic Options in Uveal Melanoma. Mol. Cancer Res..

[17] Onken M.D., Worley L.A., Long M.D., Duan S., Council M.L., Bowcock A.M., Harbour J.W. (2008). Oncogenic mutations in GNAQ occur early in uveal melanoma. Investig. Ophthalmol. Vis. Sci..

[18] Koopmans A.E., Vaarwater J., Paridaens D., Naus N.C., Kilic E., de Klein A., Rotterdam Ocular Melanoma Study group (2013). Patient survival in uveal melanoma is not affected by oncogenic mutations in GNAQ and GNA11. Br. J. Cancer.

[19] Bauer J., Kilic E., Vaarwater J., Bastian B.C., Garbe C., de Klein A. (2009). Oncogenic GNAQ mutations are not correlated with disease-free survival in uveal melanoma. Br. J. Cancer.

[20] Griewank K.G., van de Nes J., Schilling B., Moll I., Sucker A., Kakavand H., Haydu L.E., Asher M., Zimmer L., Hillen U. (2014). Genetic and clinico-pathologic analysis of metastatic uveal melanoma. Mod. Pathol..

[21] Field M.G., Durante M.A., Anbunathan H., Cai L.Z., Decatur C.L., Bowcock A.M., Kurtenbach S., Harbour J.W. (2018). Punctuated evolution of canonical genomic aberrations in uveal melanoma. Nat. Commun..

[22] Singh A.D., Zabor E.C., Radivoyevitch T. (2021). Estimating Cured Fractions of Uveal Melanoma. JAMA Ophthalmol..

[23] Szalai E., Jiang Y., van Poppelen N.M., Jager M.J., de Klein A., Kilic E., Grossniklaus H.E. (2018). Association of Uveal Melanoma Metastatic Rate With Stochastic Mutation Rate and Type of Mutation. JAMA Ophthalmol..

[24] Valsecchi M.E., Terai M., Eschelman D.J., Gonsalves C.F., Chervoneva I., Shields J.A., Shields C.L., Yamamoto A., Sullivan K.L., Laudadio M. (2015). Double-blinded, randomized phase II study using embolization with or without granulocyte-macrophage colony-stimulating factor in uveal melanoma with hepatic metastases. J. Vasc. Interv. Radiol..

[25] Patel K., Sullivan K., Berd D., Mastrangelo M.J., Shields C.L., Shields J.A., Sato T. (2005). Chemoembolization of the hepatic artery with BCNU for metastatic uveal melanoma: Results of a phase II study. Melanoma Res..

[26] Gonsalves C.F., Eschelman D.J., Adamo R.D., Anne P.R., Orloff M.M., Terai M., Hage A.N., Yi M., Chervoneva I., Sato T. (2019). A Prospective Phase II Trial of Radioembolization for Treatment of Uveal Melanoma Hepatic Metastasis. Radiology.

[27] Eschelman D.J., Gonsalves C.F., Sato T. (2013). Transhepatic therapies for metastatic uveal melanoma. Semin. Intervent. Radiol..

[28] Luscan A., Just P.A., Briand A., Burin des Roziers C., Goussard P., Nitschke P., Vidaud M., Avril M.F., Terris B., Pasmant E. (2015). Uveal melanoma hepatic metastases mutation spectrum analysis using targeted next-generation sequencing of 400 cancer genes. Br. J. Ophthalmol..

[29] McCarthy C., Kalirai H., Lake S.L., Dodson A., Damato B.E., Coupland S.E. (2016). Insights into genetic alterations of liver metastases from uveal melanoma. Pigment. Cell Melanoma Res..

[30] Yavuzyigitoglu S., Koopmans A.E., Verdijk R.M., Vaarwater J., Eussen B., van Bodegom A., Paridaens D., Kilic E., de Klein A., Rotterdam Ocular Melanoma Study G. (2016). Uveal Melanomas with SF3B1 Mutations: A Distinct Subclass Associated with Late-Onset Metastases. Ophthalmology.

[31] Van Raamsdonk C.D., Bezrookove V., Green G., Bauer J., Gaugler L., O’Brien J.M., Simpson E.M., Barsh G.S., Bastian B.C. (2009). Frequent somatic mutations of GNAQ in uveal melanoma and blue naevi. Nature.

[32] Shain A.H., Bagger M.M., Yu R., Chang D., Liu S., Vemula S., Weier J.F., Wadt K., Heegaard S., Bastian B.C. (2019). The genetic evolution of metastatic uveal melanoma. Nat. Genet..

[33] Ewens K.G., Kanetsky P.A., Richards-Yutz J., Purrazzella J., Shields C.L., Ganguly T., Ganguly A. (2014). Chromosome 3 status combined with BAP1 and EIF1AX mutation profiles are associated with metastasis in uveal melanoma. Investig. Ophthalmol. Vis. Sci..

[34] Harbour J.W., Chen R. (2013). The DecisionDx-UM Gene Expression Profile Test Provides Risk Stratification and Individualized Patient Care in Uveal Melanoma. PLoS Curr..

[35] Broggi G., Ieni A., Russo D., Varricchio S., Puzzo L., Russo A., Reibaldi M., Longo A., Tuccari G., Staibano S. (2020). The Macro-Autophagy-Related Protein Beclin-1 Immunohistochemical Expression Correlates With Tumor Cell Type and Clinical Behavior of Uveal Melanoma. Front. Oncol..

[36] Field M.G., Decatur C.L., Kurtenbach S., Gezgin G., van der Velden P.A., Jager M.J., Kozak K.N., Harbour J.W. (2016). PRAME as an Independent Biomarker for Metastasis in Uveal Melanoma. Clin. Cancer Res..

[37] van Weeghel C., Wierenga A.P.A., Versluis M., van Hall T., van der Velden P.A., Kroes W.G.M., Pfeffer U., Luyten G.P.M., Jager M.J. (2019). Do GNAQ and GNA11 Differentially Affect Inflammation and HLA Expression in Uveal Melanoma?. Cancers.

[38] Maziarz M., Leyme A., Marivin A., Luebbers A., Patel P.P., Chen Z., Sprang S.R., Garcia-Marcos M. (2018). Atypical activation of the G protein Galphaq by the oncogenic mutation Q209P. J. Biol. Chem..

[39] Amirouchene-Angelozzi N., Nemati F., Gentien D., Nicolas A., Dumont A., Carita G., Camonis J., Desjardins L., Cassoux N., Piperno-Neumann S. (2014). Establishment of novel cell lines recapitulating the genetic landscape of uveal melanoma and preclinical validation of mTOR as a therapeutic target. Mol. Oncol..

[40] Hughes S., Damato B.E., Giddings I., Hiscott P.S., Humphreys J., Houlston R.S. (2005). Microarray comparative genomic hybridisation analysis of intraocular uveal melanomas identifies distinctive imbalances associated with loss of chromosome 3. Br. J. Cancer.

[41] Parrella P., Caballero O.L., Sidransky D., Merbs S.L. (2001). Detection of c-myc amplification in uveal melanoma by fluorescent in situ hybridization. Investig. Ophthalmol. Vis. Sci..

[42] Ehlers J.P., Worley L., Onken M.D., Harbour J.W. (2005). DDEF1 is located in an amplified region of chromosome 8q and is overexpressed in uveal melanoma. Clin. Cancer Res..

