# Supplementary material for: Prognostic Values of G-Protein Mutations in Metastatic Uveal Melanoma
Source: Cancers (Basel). 2021 Nov 17;13(22):5749. doi: 10.3390/cancers13225749 (PMC8616238; doi:10.3390/cancers13225749)
Supplement: Supplementary file 1 [file cancers-13-05749-s001.zip › cancers-1393069-supplementary.pdf]

Supplementary materials: Prognostic Values of G-Protein Mutations in Metastatic Uveal Melanoma

Mizue Terai, Ayako Shimada, Inna Chervoneva, Liam Hulse, Meggie Danielson, Jeff Swensen, Marlana Orloff, Philip B. Wedegaertner, Jeffrey L. Benovic, Andrew E. Aplin and Takami Sato

Table S1. Frequency of Mutations in Metastatic Uveal Melanoma Tissues.

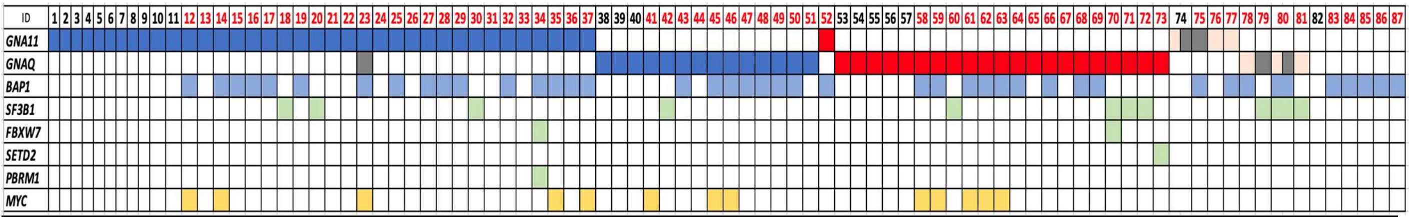

Gene Symbol	Gene Name	Genomic Location
GNA11	G protein subunit alpha 11	*19p13.3*
GNAQ	G protein subunit alpha q	*9p21.2*
BAP1	BRCA1 associated protein 1	*3p21.1*
SF3B1	splicing factor 3b subunit 1	*2p33.1*
FBXW7	F-box and WD repeat domain containing 7	*4p31.3*
SETD2	SET domain containing 2, histone lysine methyltransferase	*3p21.31*
PBRM1	polybromo 1	*3p21.1*
MYC	MYC proto-oncogene, bHLH transcription factor	*8p24.21*

Reference: HUGO Nomenclature Committee

Specimens with black ID numbers were tested for *GNA11*, *GNAQ* and *FBXW7* mutation. Specimens with red ID numbers had additional test of *BAP1* alternations, *MYC* amplification and *SF3B1/SETD2/PBRM1* mutations.
GNA11/GNAQ mutations: 209L 209P R183 Other mutations *BAP1* Alterations
SF3B1/FBXW7/ SETD2/PBRM1 mutations Amplification

Table S2. Percentage of the Estimated Tumor DNA in Specimens and Frequency of Genetic Variants.

ID	Tumor %	Gene	Variant Detail	Variant Frequency (%)
**1**	90	*GNA11*	Q209L	41
**2**	70	*GNA11*	Q209L	31
**3**	90	*GNA11*	Q209L	48
**4**	60	*GNA11*	Q209L	36
**5**	80	*GNA11*	Q209L	43
**6**	75	*GNA11*	Q209L	46
**7**	90	*GNA11*	Q209L	41
**8**	80	*GNA11*	Q209L	42
**9**	40	*GNA11*	Q209L	16
**10**	80	*GNA11*	Q209L	39
**11**	80	*GNA11*	Q209L	56
**12**	60	*GNA11*	Q209L	34
		BAP1	c.256-1G>A	50
**13**	70	*GNA11*	Q209L	31
**14**	80	*GNA11*	Q209L	35
		BAP1	R150fs	52
**15**	50	*GNA11*	Q209L	30
		BAP1	c.68-47_83del63	37
**16**	80	*GNA11*	Q209L	31
		BAP1	L108fs	39
**17**	90	*GNA11*	Q209L	39
		BAP1	T64fs	73
**18**	60	*GNA11*	Q209L	21
		SF3B1	R625H	15
**19**	80	*GNA11*	Q209L	36
		BAP1	K240fs	37
**20**	65	*GNA11*	Q209L	52
		SF3B1	N626Y	34
**21**	80	*GNA11*	Q209L	35
**22**	80	*GNA11*	Q209L	38
**23**	80	*GNA11*	Q209L	38
		GNAQ	T96S	7
		BAP1	E406*	61
**24**	60	*GNA11*	Q209L	42
**25**	70	*GNA11*	Q209L	41
		BAP1	T423fs	52
**26**	60	*GNA11*	Q209L	15
**27**	85	*GNA11*	Q209L	24
		BAP1	L108fs	34
**28**	80	*GNA11*	Q209L	28
		BAP1	H94R	38
**29**	50	*GNA11*	Q209L	9
		BAP1	K601*	51
**30**	80	*GNA11*	Q209L	25
		SF3B1	R625G	34
**31**	90	*GNA11*	Q209L	29
**32**	90	*GNA11*	Q209L	45
		BAP1	D68fs	72
**33**	80	*GNA11*	Q209L	37
**34**	80	*GNA11*	Q209L	21
		BAP1	c.784-13_802del32	51
		FBXW7	c.1856-2A>G	52

		PBRM1	c.996-1G>T	12
**35**	N/A	*GNA11*	Q209L	N/A
		BAP1	1984-1G>A	N/A
**36**	80	*GNA11*	Q209L	40
		BAP1	Q253*	66
**37**	80	*GNA11*	Q209L	38
		BAP1	P329fs	53
**38**	80	*GNAQ*	Q209L	43
**39**	90	*GNAQ*	Q209L	33
**40**	70	*GNAQ*	Q209L	16
**41**	70	*GNAQ*	Q209L	20
**42**	80	*GNAQ*	Q209L	47
		SF3B1	R625C	39
**43**	80	*GNAQ*	Q209L	18
		BAP1	Q260X	21
**44**	95	*GNAQ*	Q209L	44
**45**	90	*GNAQ*	Q209L	38
		BAP1	c.38-2_42del7	47
**46**	60	*GNAQ*	Q209L	27
		BAP1	E257fs	31
**47**	80	*GNAQ*	Q209L	42
		BAP1	G128fs	70
**48**	70	*GNAQ*	Q209L	35
		BAP1	V106fs	49
**49**	80	*GNAQ*	Q209L	45
		BAP1	R385*	86
**50**	90	*GNAQ*	Q209L	39
		BAP1	W196*	64
**51**	60	*GNAQ*	Q209L	34
**52**	70	*GNA11*	Q209P	14
		BAP1	S583fs	13
**53**	80	*GNAQ*	Q209P	70
**54**	90	*GNAQ*	Q209P	50
**55**	80	*GNAQ*	Q209P	40
**56**	80	*GNAQ*	Q209P	42
**57**	90	*GNAQ*	Q209P	38
**58**	80	*GNAQ*	Q209P	31
		BAP1	R59fs	39
**59**	30	*GNAQ*	Q209P	47
		BAP1	P149fs	81
**60**	75	*GNAQ*	Q209P	56
		SF3B1	R625C	37
**61**	90	*GNAQ*	Q209P	41
		BAP1	I643fs	71
**62**	90	*GNAQ*	Q209P	40
		BAP1	c.2057-1G>A	71
**63**	70	*GNAQ*	Q209P	41
		BAP1	c.376-32_376-3del30	69
**64**	70	*GNAQ*	Q209P	32
		BAP1	V99_S104del	46
**65**	90	*GNAQ*	Q209P	38
**66**	90	*GNAQ*	Q209P	21
		BAP1	D494fs	28
**67**	80	*GNAQ*	Q209P	24
**68**	60	*GNAQ*	Q209P	26

		BAP1	M115fs	32
**69**	90	*GNAQ*	Q209P	31
		BAP1	P175R	56
**70**	75	*GNAQ*	Q209P	28
		SF3B1	G742D	42
		FBXW7	c.585-1G>T	9
**71**	90	*GNAQ*	Q209P	34
		SF3B1	R625C	30
**72**	80	*GNAQ*	Q209P	36
		SF3B1	R625L	42
**73**	90	*GNAQ*	Q209P	48
		SETD2	c.68_71+1delCTGAG	49
**74**	85	*GNA11*	R183C	27
		GNA11	V344M	15
**75**	50	*GNA11*	Q209M	15
		BAP1	V604fs	16
**76**	20	*GNA11*	R183C	14
**77**	90	*GNA11*	R183C	42
		BAP1	Q392*	80
**78**	95	*GNAQ*	R183Q	52
		BAP1	Q712fs	46
**79**	90	*GNAQ*	G48L	60
		SF3B1	R625H	61
**80**	95	*GNAQ*	R183Q	43
		GNAQ	R338H	43
		BAP1	R385*	80
		SF3B1	R625H	42
**81**	90	*GNAQ*	R183Q	41
		SF3B1	R625C	43
**82**	80	*No mutation*		
**83**	50	*BAP1*	c.376-20_383del28	28
**84**	80	*BAP1*	C638*	92
**85**	80	*BAP1*	Y173C	47
**86**	60	*BAP1*	c.375+3_375+20del18	50
**87**	85	*BAP1*	c.375+2T>C	91

Table S3. Association between *BAP1* Alternations Status and Survival from Metastasis after Removing Patients with *SF3B1* Mutations (n = 43)

BAP1 alternation	*N* (%)	Median OS (95% CI)	*p*-Value
Yes	31 (72.0)	25.0 (19.0, 31.0)	0.845
No	12 (28.0)	22.5 (4.0, 36.0)	

Table S4. Results from the Multivariable Cox Model for OS from Met to Death in 50 Patients with Known BAP1 Alteration Status.

Factor	HR	(95% CI)	*p*-Value
Comparisons for Gene Mutation			0.010
GNA11/Q209L vs. *GNAQ*/Q209P	4.07	(1.62, 10.23)	0.003
GNAQ/Q209L vs. *GNAQ*/Q209P	3.69	(1.31, 10.36)	0.013
GNA11/Q209L vs. *GNAQ*/Q209L	1.10	(0.51, 2.40)	0.805
Age at Metastasis (Continuous)	1.03	(1.00, 1.06)	0.104
Time from Primary Tx to Metastasis (log-trans formed)	0.74	(0.56, 0.98)	0.032
BAP1 Alterations			
No	1.00	REF	
Yes	1.62	(0.75, 3.60)	0.214

Patients with *SF3B1* mutations are included in this analysis as *BAP1* negative mutations.
